# Application of ‘Readiness for Change’ concept within implementation of evidence-based mental health interventions globally: protocol for a scoping review

**DOI:** 10.12688/wellcomeopenres.18602.1

**Published:** 2022-12-05

**Authors:** Saloni Dev, Rahul Shidhaye

**Affiliations:** 1 Department of Public Health and Community Medicine, Tufts University School of Medicine, Boston, Massachusetts, 02111, USA; 2 Department of Psychiatry, Pravara Institute of Medical Sciences, Loni, Maharashtra, 413736, India; 3 Care and Public Health Research Institute, Maastricht University, Maastricht, 6200, The Netherlands

**Keywords:** Readiness for Change, Implementation Science, Mental Health, Scoping Review

## Abstract

**Background:** Concerning the growing burden of mental illnesses globally, there has been an increased investment into the implementation of evidence-based mental health interventions (EBmhIs) in routine care settings. However, the uptake and implementation of these EBmhIs has faced challenges in the real-world context. Among the many barriers and facilitators of implementation of EBmhIs identified by implementation science frameworks, evidence on the role of readiness for change (RFC) remains sparse. RFC constitutes the willingness and perceived capacity of stakeholders across an organization to implement a new practice. Theoretically, RFC has been defined at organizational, group, and individual levels, however, its conceptualization and operationalization across all these levels have differed in studies on the implementation of EBmhIs. By conducting a scoping review, we aim to examine the literature on RFC within the implementation of EBmhIs.

**Methods: **This scoping review will be conducted following the PRISMA-ScR guidelines. Iterative review stages will include a systematic and comprehensive search through four electronic databases (PubMed, Web of Science, Embase, and PsycINFO), selecting studies, charting data, and synthesizing the results. English-language studies meeting the inclusion criteria will be screened independently by two reviewers.

**Results: **This review will synthesize knowledge on the conceptualization of RFC across organizational, group, and individual levels within the implementation of EBmhIs. In addition, it will identify how RFC has been measured in these studies and summarize the reported evidence on its impact on the implementation of EBmhIs.

**Conclusions:** This review will assist mental health researchers, implementation scientists, and mental health care providers to gain a better understanding of the state of research on RFC within the implementation of EBmhIs.

**Registration:** The final protocol was registered with the Open Science Framework on October 21, 2022 ( https://osf.io/rs5n7).

## Introduction

### Background

In response to the growing burden of mental illnesses around the world, especially in light of the COVID-19 pandemic, increased attention is being paid to utilize the evidence-based mental health interventions (EBmhIs) and translate them into practice to improve the mental health outcomes ( Munoz & Cooper, 2022; Safieh *et al.*, 2022; World Health Organization, 2022). However, the actual uptake and implementation of EBmhIs in routine care settings, especially the ones with low resources has faced challenges ( Beidas *et al.*, 2021; McHugh & Barlow, 2010; Shafran *et al.*, 2009). The field of implementation science sheds further light on this *‘know-do gap’* by conceptualizing that the translation of clinical innovation into real-world settings is not spontaneous, is context-dependent, and needs methods and strategies to identify and address barriers and facilitators ( Bauer & Kirchner, 2020). When an effective EBmhI is implemented in a routine care setting, it interacts with multiple setting-specific layers such as the healthcare policies, the culture and climate of the organization implementing it, and healthcare service providers and users ( Proctor *et al.*, 2009). When these underlying interactions are not given adequate thought, there may be a risk of inadequate performance of the EBmhI or even adverse outcomes. For example, an innovative EBmhI may be perceived as a threat to the prevailing state of affairs by the service providers, which may further affect the level of adoption and acceptance of this EBmhI among them, which might, in turn, affect the fidelity with which they deliver the EBmhI.

In the last three decades, several implementation science frameworks have been developed to guide step-by-step implementation of EBmhIs and identify the barriers and facilitators in the context where they are being implemented ( Tabak *et al.*, 2012; Villalobos Dintrans *et al.*, 2019). Most of these frameworks span across multiple levels of Bronfenbrenner’s socioecological framework and posit that implementation of EBIs is influenced by factors related to the individual, organization, community, system, and policy ( Bronfenbrenner & Morris, 1998). Although a major focus of the field has been on the organizational level factors, *e.g.*, governance, leadership, resources availability, implementation climate *etc.*, scientific evidence related to the health care provider level factors, which includes readiness for change (RFC) remains sparse.

RFC constitutes the willingness and perceived capacity of stakeholders across an organization to implement a new practice ( Holt & Vardaman, 2013; Rafferty *et al.*, 2012; Vax *et al.*, 2021). Readiness for change has been conceptualized at multiple levels—at the individual level, group level, and organizational level ( Vakola, 2013). *Individual RFC* can be defined as “the extent to which an individual or individuals are cognitively and emotionally inclined to accept, embrace, and adopt a particular plan to purposefully alter the status quo”( Holt *et al.*, 2007). Individual RFC as defined here differs from the readiness for change in relation to health behaviors such as physical activity, diet, and smoking. Although both approaches draw from larger theories that explains behavior change ( Prochaska & Velicer, 1997; Rogers, 1962), the latter focuses on adoption of healthy behaviors, while the former applies to changes in the context of implementation of evidence-based interventions. Further, *group RFC* acknowledges that an individual may identify with a group within an organization such as front line workers, supervisors, or administrators. Group RFC is based on the collective perceptions and beliefs of a group that a change is needed and beneficial, and that the group and the organization have the capacity to carry out the change requirements successfully ( Vakola, 2013). Lastly, *organizational RFC* refers to macro-level factors such as organizational structure and culture, availability of resources, and leadership commitment that encourages or disrupts change ( Vakola, 2013). Although distinct, these levels do not exist in silo; they are interrelated and influence each other.

### Rationale

Considering this existence of RFC at multiple levels, it is unclear how and at what levels, RFC has been conceptualized and operationalized in the implementation of EBmhIs. Implementation of EBmhIs has previously either not considered readiness for change ( Graham *et al.*, 2020; Lyon & Bruns, 2019; Raviola *et al.*, 2019), or has popularly focused on investigating either organizational-level readiness for change ( Dorsey *et al.*, 2020; Esponda *et al.*, 2020; Myers *et al.*, 2019; Powell *et al.*, 2014; Stanhope *et al.*, 2019; Vax *et al.*, 2021), or individual readiness for change ( Hustus & Owens, 2018; Kottai & Ranganathan, 2020; Marvin & Volino Robinson, 2018; Ravitz *et al.*, 2013; Wall *et al.*, 2020). 

### Objectives

We propose to conduct a scoping review to systematically search, review, and synthesize the available evidence on the conceptualization and operationalization of RFC within the implementation of EBmhIs globally. In addition, we will also examine the reported impact of RFC on implementation of EBmhIs.

The key research questions pursued for this scoping review are:

1.   How has RFC been conceptualized within the implementation of evidence-based mental health interventions globally?

2.   How has RFC been operationalized/measured within the implementation of evidence-based mental health interventions globally?

3.   What has been the reported impact of individual-, group-, and organizational-level RFC on the implementation of evidence-based mental health interventions?

## Methodology

This scoping review will be guided by the current protocol, which has been prepared based on the methodology and reporting guidelines presented in the PRISMA-ScR extension for scoping reviews ( Tricco *et al.*, 2018). The key aspects of the review protocol are presented in this section.

### Protocol and registration

The final protocol was registered with the Open Science Framework on October 21, 2022 ( https://osf.io/rs5n7).

### Eligibility criteria

***Study designs.*** Randomized controlled trials, cohort studies, case-control studies, cross-sectional studies, case series, case reports, systematic reviews, and non-systematic/narrative reviews **will be included**.

Letters to editors, commentaries, theoretical articles, conference presentations, and chapters in textbook **will be excluded.**

***Participants.*** No limitation will be set regarding study participants as long as human subjects are involved (no animal studies).

***Intervention.*** We will include studies assessing implementation of evidence-based pharmacological/psychological/psycho-social interventions/mind-body interventions for any mental illness. There will be no limitations related to the type of intervention, length of intervention, frequency of intervention sessions or setting where the intervention is delivered (community, school, institution, *etc.*).

***Control.*** Not applicable.

***Outcome.*** The primary outcome will be readiness for change assessed at an individual, group, or organizational level.

***Timing.*** No limit will be set for timing of outcome assessments.

***Setting.*** There will be no restrictions by the type of setting. We will include studies from all geographical areas.

***Language.*** We will include articles reported in English language. A list of possibly relevant titles in other languages will be provided as an appendix.

### Information sources

The following databases will be searched: PubMed, Embase, Web of Science, and PsycINFO. Only published literature will be searched, and the literature search will be limited to the English language and human subjects. Additionally, to ensure literature saturation, forward and backward searches of included studies will be carried out, along with the advanced search in Google Scholar.

### Search strategy

The specific search strategies will be designed with the help of a librarian with expertise in searches for scoping review. First, strategy for PubMed will be developed by the research team and the librarian, and then peer reviewed by a second librarian, not otherwise associated with the project. Once the PubMed strategy is finalized, it will be adapted to the syntax and subject headings of the other databases.

A preliminary search strategy for PubMed is provided below:

((((((((((((((((((((((((((((((((((("Mental Health Services"[Mesh]) OR ("Mental Disorders"[Mesh] OR ("mental health"[All Fields]) OR ("mental illness"[All Fields])) OR ("psychological treatment"[All Fields])) OR ("depression"[All Fields])) OR ("anxiety"[All Fields])) OR ("alcohol use"[All Fields])) OR ("alcohol-use"[All Fields])) OR ("substance use"[All Fields])) OR ("substance-use"[All Fields])) OR ("substance abuse"[All Fields])) OR ("substance-abuse"[All Fields])) OR ("alcohol abuse"[All Fields])) OR ("alcohol-abuse"[All Fields])) OR ("stress"[All Fields])) OR ("post traumatic stress"[All Fields])) OR ("post-traumatic stress"[All Fields])) OR ("epilepsy"[All Fields])) OR ("suicide"[All Fields])) OR ("self harm"[All Fields])) OR ("self-harm"[All Fields])) OR ("autism"[All Fields])) OR ("dementia"[All Fields]) OR ("psychosis"[All Fields]) OR ("psychoses"[All Fields]) OR ("schizophrenia"[All Fields]) OR ("bipolar"[All Fields]) OR ("mania"[All Fields]) OR ("Depression"[Mesh]) OR ("Substance-Related Disorders"[Mesh]) OR ("Self-Injurious Behavior"[Mesh]) OR ("Psychotic Disorders"[Mesh]) OR ("Schizophrenia"[Mesh]) OR ("Depression"[Mesh]) OR ("Anxiety"[Mesh]) OR ("Alcohol-Related Disorders"[Mesh]) OR ("Trauma and Stressor Related Disorders"[Mesh]) OR "Stress Disorders, Post-Traumatic"[Mesh]) OR "Epilepsy"[Mesh]) OR "Suicide"[Mesh]) OR "Self-Injurious Behavior"[Mesh]) OR "Autistic Disorder"[Mesh]) OR "Dementia"[Mesh]) OR "Bipolar Disorder"[Mesh]) AND (("implementation"[All Fields]) OR ("Implementation Science"[Mesh])) AND ("readiness"[All Fields])) OR ("readiness for change"[All Fields])

### Data management

Literature search results from all databases will be uploaded to Endnote for data management ( *i.e.*, removing duplicates, referencing, *etc.*). They will be later exported to Rayyan software for title and abstract screening. Articles will be included if they meet our eligibility criteria.

### Selection process

Title and abstract screening will be done by an independent reviewer to select studies focused on implementation of evidence-based mental health interventions. Initially, 10% of the titles will be screened independently by SD and a research assistant, and discrepancies will be identified and resolved. This will be followed by the screening of the rest of the titles by the research assistant. Full-text screening will be done independently, SD and research assistant will select studies that meet our inclusion criteria. Full text studies that do not meet the eligibility criteria will be excluded and reasons for their exclusions will be documented in the final report. Any disagreements between the reviewers will be resolved by discussion or by including RS if no consensus can be obtained. This process will be tracked using a flow diagram, as outlined in the PRISMA-ScR guidelines.

### Data charting process

A data extraction sheet will be used by the independent reviewer to extract relevant information from the included studies. Following fields will be used to chart the data: author and date, title of the study, aim, study setting, study population, target mental illness, EBmhI utilized, level of conceptualization of readiness for change, definition of readiness for change, measure used for readiness for change, outcome, and comments. In case data is insufficient or unavailable, the authors of the studies may be contacted for clarification.

### Synthesis of results

A narrative report will be developed to summarize the extracted data around the kinds of EBmhIs, how readiness for change has been conceptualized, how it has been operationalized/measured, and its impact on the implementation of the EBmhIs. In addition, we will identify gaps in research in relation to readiness for change in implementation of EBmhIs that can be bridged by future research.

### Study status

Search terms for all the databases are finalized and we plan to perform the database search once the protocol is published.

## Discussion

RFC is a crucial implementation factor for EBmhIs. There exists a strong theoretical foundation that explains the importance of RFC, and its existence at individual, group, and organizational levels ( Vakola, 2013). Yet, it is unclear how studies on the implementation of EBmhIs conceptualize, operationalize, and measure RFC. Moreover, the evidence pool for the association of RFC with implementation outcomes is sparse. The proposed scoping review aims to synthesize knowledge to fill these gaps and inform research design, and help shape potential interventions to build RFC at multiple levels. We will note consistencies and inconsistencies in terminologies and conceptualization of RFC, how it has been measured and the psychometric properties of the assessment scales used, and the reported role of RFC on implementation of EBmhIs.

Our findings will highlight the extent to which implementation studies have conceptualized RFC at individual, group, and organizational levels. Considering the significance of RFC in the realm of implementation science ( Vakola, 2013), we posit that ideally, it is beneficial to measure RFC at all three levels in the studies of EBmhI implementation. However, we do acknowledge that not every setting globally may have the resources available for such a scale of RFC measurement. The results of our review will also identify and provide a rationale for the most important levels at which RFC may be measured depending on the setting. These findings will guide research designs to adequately conceptualize and measure RFC within the implementation of EBmhIs.

Finally, this scoping review will be pivotal for both research and practice. Researchers have discussed the conceptualization of organizational-level RFC extensively and have recommended approaches to building organizational RFC ( Domlyn *et al.*, 2021; Scaccia *et al.*, 2015; Shea *et al.*, 2014; Weiner, 2009). In addition, the role of organizational-level RFC has already been identified and promoted by prominent implementation science frameworks such as Consolidated Framework for Implementation Research (CFIR) ( Damschroder *et al.*, 2009). With this review, we aim to generate knowledge and evidence around the role of individual- and group-level RFC as well so as to build the foundation for the incorporation and integration of all levels of RFC in CFIR and other implementation science frameworks. In addition, by synthesizing knowledge around the measurement of the various levels of RFC, the results may serve as a resource to future studies aimed at developing or adapting and testing measurement instruments. This will further help health services researchers in investigating RFC as a determinant of implementation outcomes, and testing potential interventions to develop, nurture, and sustain RFC at various levels.

## Data Availability

No data are associated with this article.
