# Application of ‘Readiness for Change’ concept within implementation of evidence-based mental health interventions globally: protocol for a scoping review

**DOI:** 10.12688/wellcomeopenres.18602.2

**Published:** 2024-04-11

**Authors:** Saloni Dev, Rahul Shidhaye

**Affiliations:** 1 Department of Public Health and Community Medicine, Tufts University School of Medicine, Boston, Massachusetts, 02111, USA; 2 Care and Public Health Research Institute, Maastricht University, Maastricht, 6200, The Netherlands; 3 Department of Psychiatry, Pravara Institute of Medical Sciences, Loni, Maharashtra, 413736, India

**Keywords:** Readiness for Change, Implementation Science, Mental Health, Scoping Review

## Abstract

**Background:**

Concerning the growing burden of mental illnesses globally, there has been an increased investment into the implementation of evidence-based mental health interventions (EBmhIs) in routine care settings. However, the uptake and implementation of these EBmhIs has faced challenges in the real-world context. Among the many barriers and facilitators of implementation of EBmhIs identified by implementation science frameworks, evidence on the role of readiness for change (RFC) remains sparse. RFC constitutes the willingness and perceived capacity of stakeholders across an organization to implement a new practice. Theoretically, RFC has been defined at organizational, group, and individual levels, however, its conceptualization and operationalization across all these levels have differed in studies on the implementation of EBmhIs. By conducting a scoping review, we aim to examine the literature on RFC within the implementation of EBmhIs.

**Methods:**

This scoping review will be conducted following the PRISMA-ScR guidelines. Iterative review stages will include a systematic and comprehensive search through four electronic databases (PubMed, Web of Science, Embase, and PsycINFO), selecting studies, charting data, and synthesizing the results. English-language studies meeting the inclusion criteria will be screened independently by two reviewers.

**Discussion:**

This review will synthesize knowledge on the conceptualization of RFC across organizational, group, and individual levels within the implementation of EBmhIs. In addition, it will identify how RFC has been measured in these studies and summarize the reported evidence on its impact on the implementation of EBmhIs.

**Conclusions:**

This review will assist mental health researchers, implementation scientists, and mental health care providers to gain a better understanding of the state of research on RFC within the implementation of EBmhIs.

**Registration:**

The final protocol was registered with the Open Science Framework on October 21, 2022 ( https://osf.io/rs5n7).

## Introduction

Globally, there is a growing burden of mental illnesses, especially in light of the COVID-19 pandemic, accounting for upto 16% of global disability-adjusted life years (DALYs; approximately 418 million DALYs) ( Arias *et al.*, 2022). The Global Burden of Diseases study in 2019 also indicated that since past few decades, mental illnesses have remained among the top ten leading causes of burden worldwide ( Global Burden of Diseases (GBD) 2019 Mental Disorders Collaborators, 2022), pointing to stigma as well as limited availability of psychological treatments ( Rehm & Shield, 2019). This high burden has not only had substantial impact on all areas of life for populations, but has also costed global economy about 7.2 trillion international dollars in 2019 ( Arias *et al.*, 2022). Moreover, the World Health Organization has advocated for the importance of mental health in the achievement of sustainable development goals ( World Health Organization, 2019), emphasizing the role of mental health in overall health and well-being, encouraging economic productivity and reducing poverty, improving access to education and enhancing ability to learn, reducing violence and improving gender equality, among others ( Dybdahl & Lien, 2017).

In response to this, increased attention is being paid to utilize the evidence-based mental health interventions (EBmhIs), or psychological treatments and modes of mental health care services delivery that are supported by empirical evidence and research demonstrating their effectiveness, and translate them into practice to improve the mental health outcomes ( Muñoz & Cooper, 2022; Safieh *et al.*, 2022; World Health Organization, 2022). Considering a global point of view, testing and implementation of EBmhIs has been more prevalent in high-income countries (HICs) than in low- and middle-income countries (LMICs) ( Ingleby, 2014; Ribeiro *et al.*, 2023; Tiley & Kyriakopoulos, 2018). Notably, in all settings, the actual uptake and implementation of EBmhIs in routine care settings ( Beidas *et al.*, 2021; McHugh & Barlow, 2010; Shafran *et al.*, 2009). And this has been particularly challenging for settings that are low in resources for mental health such as LMICs where there has been scant research on the applicability and sustainability of EBmhIs (that are mostly developed in HICs) ( Ingleby, 2014; Rajabzadeh *et al.*, 2021; Ribeiro *et al.*, 2023; Tiley & Kyriakopoulos, 2018). Ultimately, the limited research and implementation has been associated with a range of factors such as lack of discussion of mental health in public health priority agenda, lack of political will, financial resources, and poor community participation, to name a few ( Tiley & Kyriakopoulos, 2018).

The field of implementation science sheds further light on this *‘know-do gap’* by clarifying that the translation of clinical innovation into real-world settings is not spontaneous, is context-dependent, and needs methods and strategies to identify and address barriers and facilitators ( Bauer & Kirchner, 2020). When an effective EBmhI is implemented in a routine care setting, it interacts with multiple setting-specific layers such as the healthcare policies, the culture and climate of the organization implementing it, and healthcare service providers and users ( Proctor *et al.*, 2009). It then becomes imperative to give these underlying interactions adequate thought, so that optimal performance of the EBmhI and favorable outcomes may be expected ( Stirman *et al.*, 2016). For example, in the context of lack of mental health professionals, large caseloads among those providing mental health care may lead to burnout or decreased quality of care ( Le *et al.*, 2022).

In the last three decades, several implementation science frameworks have been developed to guide step-by-step implementation of EBmhIs and identify the barriers and facilitators of the implementation process in the context where they are being implemented ( Tabak *et al.*, 2012; Villalobos Dintrans *et al.*, 2019). These frameworks span across multiple levels of Bronfenbrenner’s socioecological framework ( Bronfenbrenner & Morris, 1998) and posit that implementation of EBmhIs is influenced by multi-level factors ( Tabak *et al.*, 2012; Villalobos Dintrans *et al.*, 2019). Table 1 lists some examples at multiple levels that have been reported to influence implementation.

**Table 1.** Examples of Factors Influencing Implementation of EBImhIs at Multiple Levels.

Domains/Levels	Inexhaustive Examples of Barriers and Facilitators ( Babatunde *et al.*, 2021; Le *et al.*, 2022; March *et al.*, 2022; Troup *et al.*, 2021; Webb *et al.*, 2021)
Individual (patient, staff, healthcare providers)	Motivation, skills, engagement, training, supervision
Intervention	Content, design, alignment with needs, cost, timing, duration,
Organization	Leadership, communication, culture, resources availability
Community and Society	Sociocultural norms, economic conditions, stigma, religion/spirituality
Health Care System and Governance	Governmental and bureaucratic support, infrastructure, policy, funding

Notably, what remains sparse is a focus on readiness for change (RFC), which cuts across multiple levels and represents a complex interaction between these levels, as we explain below.

RFC constitutes the willingness and perceived capacity of stakeholders across an organization to implement a new practice ( Holt & Vardaman, 2013; Rafferty *et al.*, 2012; Vax *et al.*, 2021). Readiness for change has been conceptualized at multiple levels—at the individual level, group level, and organizational level ( Vakola, 2013). *Individual RFC* can be defined as “the extent to which an individual or individuals are cognitively and emotionally inclined to accept, embrace, and adopt a particular plan to purposefully alter the status quo” ( Holt *et al.*, 2007). Individual RFC as defined here differs from the readiness for change in relation to health behaviors such as physical activity, diet, and smoking. Although both approaches draw from larger theories that explains behavior change ( Prochaska & Velicer, 1997; Rogers, 1962), the latter focuses on adoption of healthy behaviors, while the former applies to changes in the context of implementation of evidence-based interventions. Further, *group RFC* acknowledges that an individual may identify with a group within an organization such as front line workers, supervisors, or administrators. Group RFC is based on the collective perceptions and beliefs of a group that a change is needed and beneficial, and that the group and the organization have the capacity to carry out the change requirements successfully ( Vakola, 2013). Lastly, *organizational RFC* refers to macro-level factors such as organizational structure and culture, availability of resources, and leadership commitment that encourages or disrupts change ( Vakola, 2013). Ultimately, when organizational readiness is high, it exerts a favorable influence on implementation of interventions via organization members’ inclination to change, exert greater effort, and cooperate with others ( Weiner, 2009). Although distinct, these levels do not exist in silo; they are interrelated and influence each other.

Considering this existence of RFC at multiple levels, it is unclear how and at what levels, RFC has been conceptualized and operationalized in the implementation of EBmhIs. Implementation of EBmhIs has previously either not considered readiness for change ( Graham *et al.*, 2020; Lyon & Bruns, 2019; Raviola *et al.*, 2019), or has popularly focused on investigating either organizational-level readiness for change ( Dorsey *et al.*, 2020; Esponda *et al.*, 2020; Myers *et al.*, 2019; Powell *et al.*, 2014; Stanhope *et al.*, 2019; Vax *et al.*, 2021), or individual readiness for change ( Hustus & Owens, 2018; Kottai & Ranganathan, 2020; Marvin & Volino Robinson, 2018; Ravitz *et al.*, 2013; Wall *et al.*, 2020). The proposed scoping review aims to refine and build upon a previous study that synthesized the conceptualization and measurement of organization-level readiness for change in health services research ( Weiner *et al.*, 2008). This review specifically focuses on the implementation of EBmhIs and profiles the various levels at which readiness for change has been conceptualized.

### Objectives

We propose to conduct a scoping review to systematically search, review, and synthesize the available evidence on the conceptualization and operationalization of RFC within the implementation of EBmhIs globally. In addition, we will also examine the reported impact of RFC on implementation of EBmhIs.

The key research questions pursued for this scoping review are:

1.   How has RFC been conceptualized within the implementation of EBmhIs globally?

2.   How has RFC been operationalized/measured within the implementation of EBmhIs globally?

3.   What has been the reported impact of individual-, group-, and organizational-level RFC on the implementation of EBmhIs (particularly, how RFC influences key implementation outcomes such as adoption, feasibility, fidelity, and sustainability of EBmhIs)?

## Methodology

This scoping review will be guided by the current protocol, which has been prepared based on the methodology and reporting guidelines presented in the Preferred Reporting Items for Systematic reviews and Meta-Analyses extension for Scoping Reviews (PRISMA-ScR) ( Tricco *et al.*, 2018). The key aspects of the review protocol are presented in this section.

### Protocol and registration

The final protocol was registered with the Open Science Framework on October 21, 2022 ( https://osf.io/rs5n7).

### Eligibility criteria

***Study designs.*** Randomized controlled trials, cohort studies, case-control studies, cross-sectional studies, case series, case reports, systematic reviews, and non-systematic/narrative reviews **will be included**.

Letters to editors, commentaries, theoretical articles, conference presentations, and chapters in textbook **will be excluded.** Typically, these articles serve a different purpose than those outlining empirical studies, and do not lend themselves to analysis that would align with our research questions.

***Population.*** We will include studies involving human subjects of any age. There will be no limitations on the specific characteristics or demographics of the participants.

***Concept.*** We will include studies assessing the implementation of evidence-based pharmacological, psychological, psycho-social, or mind-body interventions for any mental illness. There will be no limitations related to the type, duration, frequency, or delivery setting of the interventions (community, school, institution, etc.).

***Context.*** There will be no limitations on the context of the studies, and interventions delivered in various settings (community, school, institution, etc.) will be eligible for inclusion.

***Timing.*** No limit will be set for timing of outcome assessments.

***Setting.*** There will be no restrictions by the type of setting. We will include studies from all geographical areas.

***Language.*** We will include articles reported in English language. A list of possibly relevant titles in other languages will be provided as an appendix.

### Information sources

The following databases will be searched: PubMed, Embase, Web of Science, and PsycINFO. Only published literature will be searched, and the literature search will be limited to the English language and human subjects. Additionally, to ensure literature saturation, forward and backward searches of included studies will be carried out, along with the advanced search in Google Scholar. We will exclude grey literature from our search given the wide range of sources it encompasses and in our attempt to prioritize peer-reviewed studies that have undergone critical assessment. We will limit our search to articles published until November 2022.

### Search strategy

The specific search strategies will be designed with the help of a librarian with expertise in searches for scoping review. First, strategy for PubMed will be developed by the research team and the librarian, and then peer reviewed by a second librarian, not otherwise associated with the project. Once the PubMed strategy is finalized, it will be adapted to the syntax and subject headings of the other databases.

A preliminary search strategy for PubMed is provided below:

((((((((((((((((((((((((((((((((((("Mental Health Services"[Mesh]) OR ("Mental Disorders"[Mesh] OR ("mental health"[All Fields]) OR ("mental illness"[All Fields])) OR ("psychological treatment"[All Fields])) OR ("depression"[All Fields])) OR ("anxiety"[All Fields])) OR ("alcohol use"[All Fields])) OR ("alcohol-use"[All Fields])) OR ("substance use"[All Fields])) OR ("substance-use"[All Fields])) OR ("substance abuse"[All Fields])) OR ("substance-abuse"[All Fields])) OR ("alcohol abuse"[All Fields])) OR ("alcohol-abuse"[All Fields])) OR ("stress"[All Fields])) OR ("post traumatic stress"[All Fields])) OR ("post-traumatic stress"[All Fields])) OR ("epilepsy"[All Fields])) OR ("suicide"[All Fields])) OR ("self harm"[All Fields])) OR ("self-harm"[All Fields])) OR ("autism"[All Fields])) OR ("dementia"[All Fields]) OR ("psychosis"[All Fields]) OR ("psychoses"[All Fields]) OR ("schizophrenia"[All Fields]) OR ("bipolar"[All Fields]) OR ("mania"[All Fields]) OR ("Depression"[Mesh]) OR ("Substance-Related Disorders"[Mesh]) OR ("Self-Injurious Behavior"[Mesh]) OR ("Psychotic Disorders"[Mesh]) OR ("Schizophrenia"[Mesh]) OR ("Depression"[Mesh]) OR ("Anxiety"[Mesh]) OR ("Alcohol-Related Disorders"[Mesh]) OR ("Trauma and Stressor Related Disorders"[Mesh]) OR "Stress Disorders, Post-Traumatic"[Mesh]) OR "Epilepsy"[Mesh]) OR "Suicide"[Mesh]) OR "Self-Injurious Behavior"[Mesh]) OR "Autistic Disorder"[Mesh]) OR "Dementia"[Mesh]) OR "Bipolar Disorder"[Mesh]) AND (("implementation"[All Fields]) OR ("Implementation Science"[Mesh])) AND ("readiness"[All Fields])) OR ("readiness for change"[All Fields])

### Data management

Literature search results from all databases will be uploaded to Endnote for data management ( *i.e.*, removing duplicates, referencing, *etc.*). They will be later exported to Rayyan software for title and abstract screening. Articles will be included if they meet our eligibility criteria.

### Selection process

**Figure 1.**
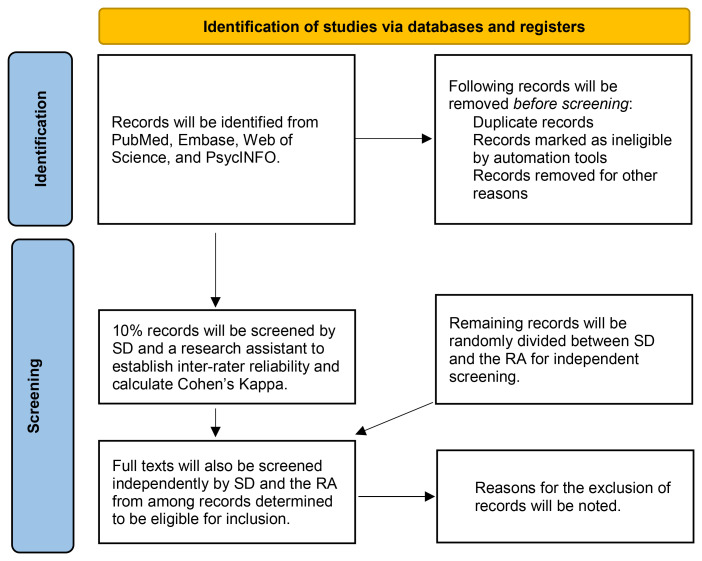
PRISMA Flow Diagram Adapted to Explain Our Selection Process.

Title and abstract screening will be done by an independent reviewers to select studies focused on implementation of evidence-based mental health interventions. Initially, ~50 titles will be utilized by SD and a research assistant (RA) to practice applying the eligibility criteria, and discrepancies will be identified and resolve. Next, 10% of the titles will be screened independently by both SD and the RA, to calculate inter-rater reliability using Cohen’s Kappa. Any disagreements between the reviewers will be resolved by discussion or by including RS if no consensus can be obtained. Once an acceptable value of Cohen’s Kappa is obtained (>80%), the rest of the titles will be randomly divided into two sets that will be screened by SD and RA individually. Full-text screening of the included titles will be done by the RA. Full text studies that do not meet the eligibility criteria will be excluded and reasons for their exclusions will be documented in the final report. This process will be tracked using a flow diagram, as outlined in the PRISMA-ScR guidelines ( Page *et al.*, 2021), which we have modified in Figure 1 to visually depict our selection process.

### Data charting process

A data extraction sheet will be used by the independent reviewer to extract relevant information from the included studies. Following fields will be used to chart the data: author and date, title of the study, aim, study setting, study population, target mental illness, EBmhI utilized, level of conceptualization of readiness for change, definition of readiness for change, measure used for readiness for change, outcome, and comments. In case data is insufficient or unavailable, the authors of the studies may be contacted for clarification.

### Synthesis of results

A narrative report will be developed to summarize the extracted data around the kinds of EBmhIs, how readiness for change has been conceptualized, how it has been operationalized/measured, and its impact on the implementation of the EBmhIs. In addition, we will identify gaps in research in relation to readiness for change in implementation of EBmhIs that can be bridged by future research.

### Study status

Search terms for all the databases are finalized and we plan to perform the database search once the protocol is published.

## Discussion

RFC may be a crucial implementation factor for EBmhIs. There exists a strong theoretical foundation that explains the importance of RFC, and its existence at individual, group, and organizational levels ( Vakola, 2013). Yet, it is unclear how studies on the implementation of EBmhIs conceptualize, operationalize, and measure RFC. Moreover, the evidence pool for the association of RFC with implementation outcomes is sparse. The proposed scoping review aims to synthesize knowledge to fill these gaps and inform research design, and help shape potential interventions to build RFC at multiple levels. We will note consistencies and inconsistencies in terminologies and conceptualization of RFC, how it has been measured and the psychometric properties of the assessment scales used, and the reported role of RFC on implementation of EBmhIs.

Our findings will highlight the extent to which implementation studies have conceptualized RFC at individual, group, and organizational levels. Considering the significance of RFC in the realm of implementation science ( Vakola, 2013), we posit that ideally, it is beneficial to measure RFC at all three levels in the studies of EBmhI implementation. However, we do acknowledge that not every setting globally may have the resources available for such a scale of RFC measurement. Based on the evidence gathered to address our third research question regarding the influence of RFC on the implementation of EBmhIs, our review aims to identify and provide a rationale for the key levels at which RFC should be measured, depending on the specific setting. These findings will guide research designs to adequately conceptualize and measure RFC within the implementation of EBmhIs.

Finally, this scoping review will be pivotal for both research and practice. Researchers have discussed the conceptualization of organizational-level RFC extensively and have recommended approaches to building organizational RFC ( Domlyn *et al.*, 2021; Scaccia *et al.*, 2015; Shea *et al.*, 2014; Weiner, 2009). In addition, the role of organizational-level RFC has already been identified and promoted by prominent implementation science frameworks such as Consolidated Framework for Implementation Research (CFIR) ( Damschroder *et al.*, 2009). With this review, we aim to generate knowledge and evidence around the role of individual- and group-level RFC as well so as to build the foundation for the incorporation and integration of all levels of RFC in CFIR and other implementation science frameworks. In addition, by synthesizing knowledge around the measurement of the various levels of RFC, the results may serve as a resource to future studies aimed at developing or adapting and testing measurement instruments. This will further help health services researchers in investigating RFC as a determinant of implementation outcomes, and testing potential interventions to develop, nurture, and sustain RFC at various levels.

### Expected strengths and limitations

To the best of our knowledge, this will be the first scoping review examining readiness for change within the implementation of EBmhIs globally. The results we report would need to be considered in light of certain limitations. First, readiness for change has been subject to the jingle fallacy i.e., many different terms has been used to refer to the same concept (for example, preparedness, willingness, commitment, openness, etc.) ( Miake-Lye *et al.*, 2020). However, our initial attempt to include such related terms in our search strategy led to a number of results that were unmanageable. Hence, we may have missed relevant studies in an attempt to focus our search strategy. Furthermore, we did not conduct a search of gray literature, and as such, the conclusions we’ll draw are limited in generalizability. Finally, the inclusion of studies published in English may also limit the generalizability of the results we report.

### Implications for future research

Overall, this scoping review may also hold potential to guide future research. By highlighting the varying conceptualizations as well as operationalization of RFC, future studies could advocate for and use consistent, unified framework for RFC in the context of implementing EBmhIs. This could involve synthesizing existing definitions and developing a more standardized way to measure RFC across different levels. In the longer term, such would also enhance the feasibility of conducting comprehensive systematic reviews and meta-analyses. In addition, this review could also motivate longitudinal studies to provide insights into the dynamic nature of readiness, identifying how readiness might impact implementation success.

## Data Availability

No data are associated with this article.
