# Supplementary material for: Application of ‘Readiness for Change’ concept within implementation of evidence-based mental health interventions globally: protocol for a scoping review
Source: Wellcome Open Res. 2024 Apr 11;7:293. Originally published 2022 Dec 5. [Version 2] doi: 10.12688/wellcomeopenres.18602.2 (PMC10314182; doi:10.12688/wellcomeopenres.18602.2)
Supplement: Peer review history

# Reviewer report: Reviewer response for version 1

**DOI:** 10.21956/wellcomeopenres.20628.r61242

**Published:** 2023-07-07

**Authors:** Prerna Banati

**Affiliations:** 1 World Health Organization, Geneva, Geneva, Switzerland

Overall this is worthwhile study to map RFC to IR concepts. The research question is clear and the study methodology is sound. Some suggestions here offered:

1. Consider whether it might be feasible to review grey literature, even in a small selection of countries. I imagine many of these implementation considerations may not be published in the peer reviewed literature, but rather sit within implementers manuals or local monitoring reports. Even a deep dive in a few countries may provide a useful contribution to scoping. 
2. Mental health interventions are a broad group, and wonder if it might be sensible to narrow down to a more manageable category. For example: MH interventions for children, or those delivered only in primary care settings. 
3. The publication dates for eligible papers is not defined and should ideally be included in the methodology.

Is the study design appropriate for the research question?

Yes

Is the rationale for, and objectives of, the study clearly described?

Yes

Are sufficient details of the methods provided to allow replication by others?

Yes

Are the datasets clearly presented in a useable and accessible format?

Yes

Reviewer Expertise:

Implementation research, Adolescents, Mental health,

I confirm that I have read this submission and believe that I have an appropriate level of expertise to confirm that it is of an acceptable scientific standard.

# Reviewer report: Reviewer response for version 1

**DOI:** 10.21956/wellcomeopenres.20628.r59954

**Published:** 2023-06-30

**Authors:** Emmanuel Biracyaza

**Affiliations:** 1 Centre for Interdisciplinary Research in Rehabilitation of Greater Montreal (CRIR), Montreal, Québec, Canada

**Report:**

 I believe this paper is valuable offering a significant perspective on a topic that has not, as yet, had much clarity. In general, the manuscript is well written and provides crucial information for the field. I commend the authors for their work. The protocol introduced in this paper initiates a discussion that has the potential to advance the field of implementation science. The proposed review aims to examine the existing scientific literature regarding the conceptualization and measurement of readiness for change in the implementation of evidence-based mental health interventions across various levels, including organizational, group, and individual. Below, I have provided some comments that may assist in refining the key messages of the paper and distinguishing it more effectively from existing reviews in this domain.

 I believe this paper requires a thorough proofreading. Several important sentences require supporting evidence from previous studies. I recommend reviewing the background section for accuracy and completeness.

**Abstract:**

 The abstract of this protocol is well written, however, I do not agree with the authors that they can include results. As the data collection and analyses were not performed, I recommend the authors to use “Discussion” instead of the “results” on the first page.

**Background**:

 The authors mentioned that there are various implementation science frameworks available to guide the step-by-step implementation of evidence-based mental health interventions (EBmhIs) and to identify the barriers and facilitators within specific implementation contexts. To provide more examples of factors that enable and hinder the implementation of such frameworks, the authors could explore additional studies or literature that discuss specific facilitators and barriers. This could include factors such as organizational support, stakeholder engagement, resource availability, training and capacity building, cultural considerations, leadership buy-in, and financial constraints, among others.

 In the background section, the authors initially mentioned that mental disorders are burdensome, but they did not specify such mental disorders and their prevalence. To address this, the authors can include relevant statistics or research findings that highlight the prevalence and impact of specific mental disorders, such as major depressive disorder, on a global scale. They could elaborate on how these disorders contribute to the burden by discussing their effects on quality of life, disability-adjusted life years (DALYs), or quality-adjusted life years (QALYs), and other relevant measures.

 Moreover, to establish a connection between mental disorders and the achievement of sustainable development goals (SDGs), the authors could provide evidence or references that demonstrate how mental disorders act as barriers to the attainment of SDGs. They can explore studies or reports that showcase the impact of mental health issues on various dimensions of sustainable development, such as poverty reduction, education, gender equality, economic productivity, and social cohesion. This would strengthen the justification for the importance of addressing mental disorders within the context of the SDGs.

 Considering that the study was conducted at the global level, it is essential for the authors to provide information on the implementation of evidence-based mental health interventions (EBmhIs) in different types of countries, including low- and middle-income countries, high-income countries, and middle-income countries. They should explain whether EBmhIs are well-known and widely implemented in these countries, and if not, what are the obstacles and barriers hindering their implementation.

 More literature on conceptualisation of organisational readiness for change is needed.

 How is your study different from the previous studies especially scoping review, systematic and metaanalyses studies? What is the novelty of your study?

 I observe that the authors describe a theory of organisational readiness for change, which refers to an organizational-level construct referring to “organizational member’s shared resolve to implement a change (change commitment) and shared belief in their collective capability do to do (change efficacy).” So, I think that the authors have to clarify more details about organisational reediness change. They also have to critique available instruments for measuring organizational readiness for change as having “limited evidence of reliability and validity.”

 The rationale is the justification for undertaking a study in a particular population and areas of interest. Based on the information provided by the authors in the subheading “rationale”, the text provided seems unclear for me or the text does not fit in the subheading. So, I can suggest the authors to remove the subheading or strengthen rationale by explain the reason why they conduct this scoping study (clarifying a research gap and justification).

**Objective**: The research questions do not cover well the EBmhIs, can this be clarified if they are different from RFC?

**Methods:**

 The study was conducted in on EBmhIs which is mental health domain. This indicates that there many databases such as MEDLINE, Scopus, EBSCO, Science Direct, Embase, CINAHL, PubMed, Web of Sciences, PsycInfo, Cochrane, Web of Science, and Maestro and in ProQuest which are appropriate to find potential studies. But the authors selected only 4 databases. Why do you not include these databases which have many publications on mental health?

**Search strategy**: The terms used to select potential studies for scoping review are not enough. This is because the readiness for change is related to several terms such as openness to change, attitudes or behaviors toward change, commitment to organisational change.

 On page 4, the authors said that “Letters to editors, commentaries, theoretical articles, conference presentations, and chapters in textbook will be excluded”, please, you have to explain why you excluded them. This is helpful for the readers.

**Discussion**: In the discussion section you say that the results will help to “identify and provide a rationale for the most important levels at which RFC may be measured depending on the setting”. It is not completely clear to me how the review will answer this question. Do you mean at which levels it has been most frequently measured in different settings?

 I think that you should provide the following information; (1) expected strengths and limitations, and (2) public health implications / recommendations to the further research. 

**Minor errors:**

- Please, on page 2 (line 1 and line 2), you cannot use subheading at the start of your main text “background and introduction”.
- You need a reference on page 2 at the end of the first of the first paragraph / in the background.
- Please, provide a reference at the end of the second paragraph of the background.
- Before you start using PRISMA, please, provide its full words.

Is the study design appropriate for the research question?

Yes

Is the rationale for, and objectives of, the study clearly described?

No

Are sufficient details of the methods provided to allow replication by others?

Partly

Are the datasets clearly presented in a useable and accessible format?

Partly

Reviewer Expertise:

Mental health, Family health, Parenting, Child health, Occupation science

I confirm that I have read this submission and believe that I have an appropriate level of expertise to confirm that it is of an acceptable scientific standard.

# Reviewer report: Reviewer response for version 1

**DOI:** 10.21956/wellcomeopenres.20628.r59101

**Published:** 2023-06-30

**Authors:** John Broughan

**Affiliations:** 1 University College Dublin, Dublin, Leinster, Ireland

Thank you for asking me to review this manuscript. It is very well constructed and written. There are some things worth reconsidering and revising but these are minor. I have outlined my feedback below in relation to the study aims, rationale, and methods. I also agree with the previous reviewer’s feedback which I read after completing my own review.

**Aim:** The study aims outlines a protocol for a scoping review. The proposed scoping review aims to examine literature on the concept of Readiness for Change (RFC) as it relates to evidence-based mental health interventions (EBmhIs).

**Rationale for the area of study:** The authors say that research regarding RFC in EBmhIs contexts is limited. Thus, the proposed review aims to address this issue and fill existing knowledge gaps regarding RFC’s conceptualisation, operationalisation, and impact in EBmhIs. The rationale is sound and is consistent with the proposed review’s outlined objectives, which are communicated clearly.

**Method:** A scoping review method will be used (which is appropriate for an exploratory study like this) and will appropriately be guided by the PRISMA ScR guidelines. The outlined protocol has done a good job of structuring the scoping review in line with these guidelines.

 Additional methodological guidance from established scoping review frameworks (e.g., Arksey & O’Malley, 2005 ^1^; Peters *et al.*, 2020 ^2^) could also be useful and is worth consulting if this hasn’t been done. Having said this, adherence to these frameworks is not essential as the PRISMA ScR is comprehensive and informed by them anyway.

 The eligibility criteria should contain details on the publication year range that will be included (if any).

 There should be a brief rationale for why grey literature (e.g., letters to editors) is not to be included.

 The eligibility criteria list is partly informed by the PICO framework. This is not incorrect, but it is also worth considering how the PCC (Population, Concept, Context) framework might influence the criteria. The PCC model is recommended for scoping reviews by the Joanna Briggs Institute.

 Some detail could be added to explain things more clearly in the Selection Process section. Adding a PRISMA flowchart outlining the process might make things clearer.

**Discussion:** The first line says that “RFC is a crucial implementation factor for EBmhIs…”. This struck me as a problematic claim. My thinking was that you will need to complete your scoping review to decide whether RFC is a crucial factor in this context. Perhaps rephrasing to say “RFC *may* be a crucial implementation factor…” or consider deleting the line?

Is the study design appropriate for the research question?

Yes

Is the rationale for, and objectives of, the study clearly described?

Yes

Are sufficient details of the methods provided to allow replication by others?

Partly

Are the datasets clearly presented in a useable and accessible format?

Not applicable

Reviewer Expertise:

Healthcare services research; primary care' general practice; mental health, Covid-19 (Long Covid)

I confirm that I have read this submission and believe that I have an appropriate level of expertise to confirm that it is of an acceptable scientific standard.

## References

[1] : Scoping studies: towards a methodological framework. *International Journal of Social Research Methodology: Theory* .

[2] : Chapter 11: Scoping Reviews (2020 version). In: Aromataris E, Munn Z (Editors). JBI Manual for Evidence Synthesis. *JBI* .2020; Reference source

# Reviewer report: Reviewer response for version 1

**DOI:** 10.21956/wellcomeopenres.20628.r53612

**Published:** 2022-12-20

**Authors:** Hanna Augustsson

**Affiliations:** 1 Procome Research Group, Department of Learning, Informatics, Management and Ethics, Medical Management Centre, Karolinska Institutet, Stockholm, Sweden

Thank you for the opportunity to review this scoping review protocol. The proposed review will scope the scientific literature on the conceptualization and measurement of readiness for change for implementation of EBmhIs across organizational, group, and individual levels. The study protocol is outlined according to the Prisma-Scr and the authors provide a clear description of the planned methods that will be used in the different stages in the review process. I have some minor comments and suggestions.

 Background: Weiner et al have conducted a review of the literature around the conceptualization and measurement of organizational readiness for change in 2008. Although it is now quite old and not limited to mental health, I recommend that you mention it in your protocol and/or in your scoping review with description about how your review differ from/add to this review. (Weiner BJ, Amick H, Lee SY. Conceptualization and measurement of organizational readiness for change: a review of the literature in health services research and other fields. Med Care Res Rev. 2008 Aug;65(4):379-436 ^1^)

 Objective: It is not completely clear what type of impact you will assess in RQ3, can this be clarified? 

 Search strategy: There may be other terms that have been used to describe readiness for change, e.g. readiness for organizational change (Armenakis et al., 1993 ^2^), organizational readiness for change (Weiner et al., 2008) which you should be able to capture with your suggested search strategy. My question concerns other related terms such as commitment to organizational change (Herscovitch & Meyer, 2002 ^3^; Michaelis, Stegmaier, & Sonntag, 2010 ^4^), receptivity to change (Frahm & Brown, 2007 ^5^) and attitudes toward change (Elias, 2007 ^6^; Lines, 2005 ^7^) or openness to change. These constructs have sometimes been used interchangeably with readiness for change and you may want to consider adding related terms to the search strategy.

 Discussion: In the discussion section you say that the results will help to “identify and provide a rationale for the most important levels at which RFC may be measured depending on the setting”. It is not completely clear to me how the review will answer this question. Do you mean at which levels it has been most frequently measured in different settings?

Is the study design appropriate for the research question?

Yes

Is the rationale for, and objectives of, the study clearly described?

Yes

Are sufficient details of the methods provided to allow replication by others?

Yes

Are the datasets clearly presented in a useable and accessible format?

Not applicable

Reviewer Expertise:

implementation science

I confirm that I have read this submission and believe that I have an appropriate level of expertise to confirm that it is of an acceptable scientific standard.

## References

[1] : Conceptualization and measurement of organizational readiness for change: a review of the literature in health services research and other fields. *Med Care Res Rev* .2008;65(4) : 10.1177/1077558708317802 379-436 10.1177/1077558708317802 18511812

[2] : Creating Readiness for Organizational Change. *Human Relations* .1993;46(6) : 10.1177/001872679304600601 681-703 10.1177/001872679304600601

[3] : Commitment to organizational change: extension of a three-component model. *J Appl Psychol* .2002;87(3) : 10.1037/0021-9010.87.3.474 474-87 10.1037/0021-9010.87.3.474 12090605

[4] : Shedding light on followers' innovation implementation behavior. *Journal of Managerial Psychology* .2010;25(4) : 10.1108/02683941011035304 408-429 10.1108/02683941011035304

[5] : First steps: linking change communication to change receptivity. *Journal of Organizational Change Management* .2007;20(3) : 10.1108/09534810710740191 370-387 10.1108/09534810710740191

[6] : Employee Commitment in Times of Change: Assessing the Importance of Attitudes Toward Organizational Change †. *Journal of Management* .2009;35(1) : 10.1177/0149206307308910 37-55 10.1177/0149206307308910

[7] : How social accounts and participation during change affect organizational learning. *Journal of Workplace Learning* .2005;17(3) : 10.1108/13665620510588680 157-177 10.1108/13665620510588680
